# Triplanar Chevron Osteotomy: A Newly Proposed Method to Treat Hallux Valgus Deformities

**DOI:** 10.1111/os.12806

**Published:** 2020-10-19

**Authors:** Jia‐liang Guo, Wei‐chong Dong, Mei‐shuang Shang, Kuo Zhao, Jun‐yong Li, Zhi‐yong Hou, Ying‐ze Zhang

**Affiliations:** ^1^ Trauma Centre, Department of Orthopaedics The Third Hospital of Hebei Medical University Shijiazhuang China; ^2^ Clinical Pharmacy, Department of Pharmacy The Second Hospital of Hebei Medical University Shijiazhuang China; ^3^ Chinese Academy of Engineering Beijing China

**Keywords:** Chevron osteotomy, Hallux valgus, Mimics, Triplanar deformity

## Abstract

**Objective:**

To present a novel method called triplanar chevron osteotomy to treat hallux valgus (HV).

**Methods:**

This is a retrospective study. In this study, the CT data of HV patients with painful callosities were evaluated retrospectively between 1 June 2018 and 1 June 2020. CT data from 49 consecutive patients (59 feet) with HV were evaluated. The average age at the time of surgery was 49.6 years (range, 30–63 years). The apex of the chevron osteotomy procedure was located at the center of the first metatarsal and was defined as the line formed by the central point perpendicular to the fourth metatarsal bone. The cut planes of the plantarward oblique chevron osteotomy (POCO) were defined as follows: chevron osteotomy along with 20° of plantarward obliquity. The triplanar osteotomy incision was made using the POCO method, with the direction inclined by 10° distally. The intermetatarsal angle (IMA), the HV angle (HVA), the projection of the second metatarsal (PSM), the metatarsal protrusion index (MPI), and the metatarsal protrusion distance (MPD) were all calculated before and after the operations. The length of the first metatarsal was measured and calculated with an equation.

**Results:**

The results showed that the HVA was significantly decreased after surgery (32.7° ± 4.6° *vs* 14.9° ± 2.1°, *t* = 25.583, *P* < 0.001) in the triplanar, traditional, and POCO groups. The IMA was also significantly decreased (14.7° ± 2.0°) compared with the results before surgery (8.0° ± 1.1°, *t* = 22.739, *P* < 0.001) in these groups. Compared with traditional osteotomy and POCO, there were no differences in correcting deformities on axial planes for the HVA (14.5° ± 1.7° *vs* 14.9° ± 2.1°, *t* = 1.835, *P* = 0.072) and IMA (8.1° ± 1.1° *vs* 8.0° ± 1.1°, *t* = −0.97, *P* = 0.336). There was a statistically significant decrease following surgery in terms of the PSM, MPI, and MPD after triplanar osteotomy. The length of the first metatarsal increased (10.9 ± 1.3 mm), as measured through three‐dimensional images in the triplanar osteotomy group. The length was calculated as follows: H = L2 * Tan θ ≈ L/COS β * Tan θ.

**Conclusion:**

The new triplanar osteotomy technique is safe and effective for treating HV, and in simulation experiments reveals potential benefits of correction and preventing transfer metatarsalgia.

## Graphical abstract

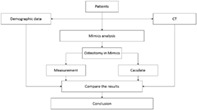

The aim of this research was to present a novel method called triplanar chevron osteotomy to treat hallux valgus (HV). CT data from 49 consecutive patients (59 feet) with HV were evaluated. The intermetatarsal angle (IMA), the HV angle (HVA), the projection of the second metatarsal (PSM), the metatarsal protrusion index (MPI), and the metatarsal protrusion distance (MPD) were all calculated before and after the operations. The length of the first metatarsal was measured and calculated with an equation. The length of the first metatarsal increase was calculated as follows: H = L2 * Tan θ ≈ L/COS β * Tan θ. Therefore, the new triplanar osteotomy technique is safe and effective for treating HV, and in simulation experiments reveals potential benefits of correction and preventing transfer metatarsalgia.

## Introduction

Hallux valgus (HV), with a prevalence ranging from 21% to 70%, is a deformity with abduction of the first metatarsophalangeal along with rotation of the first toe and lateral deviation^1^, ^2^, ^3^. Chevron osteotomy is a surgical procedure used in mild or moderate HV patients. However, HV is regarded as having a multifactorial origin. To improve the effect of treatment, different variants have been proposed over time. Most studies have focused on evaluating the deformity in one plane, and the traditional operations for HVA correction address uniplanar deformity^4^, ^5^.

As we mentioned in our previous research, HV is a deformity represented in three different planes (coronal, sagittal, and transverse planes). We proposed a modified plantarward oblique chevron osteotomy (POCO) procedure to increase the plantar migration of the metatarsal head; it was proven to be a safe and effective method to treat HV and to offer potential benefits through correction and prevention of transfer metatarsalgia^6^. In addition, the modified POCO procedure corrected the deformities in the coronal and sagittal planes.

Deformities in the transverse or axial planes are categorized by the length of the first metatarsal bone. Shortening of the first ray is considered the main cause of pain and results in transfer metatarsalgia^7^. Thus, most surgeons tend not to shorten the first metatarsal regardless of the procedures they choose. However, although biplanar‐modified POCO is a suitable and optional method to treat HV with painful plantar callosities, it cannot preserve the length of the first metatarsal, which is essential for the maintenance of weight‐bearing. The aim of the research was: (i) to present a novel method and try to improve the treatment effect of HV; (ii) to mimic operation procedures within software; and (iii) to compare the novel method with traditional methods proposed by other authors.

## Materials and Methods

### *Inclusion and Exclusion Criteria*

In this study, the CT data of HV patients with painful callosities were evaluated retrospectively between 1 June 2018 and 1 June 2020. Informed consent was obtained by telephone from all patients enrolled in the study. Patients' motivation for receiving operations was dissatisfaction with conservative treatment, such as hallux‐straightening devices, footwear modifications, and other physiotherapies. All patients were treated by one experienced surgeon in our department (SJ Q).

Inclusion criteria for the study were as follows: (i) whole imaging data for measurement; (ii) clear record of demographic data; (iii) a retrospective study; and (iv) three‐dimensional construction with Mimics.

Exclusion criteria for the study were illustrated as follows: (i) patients with calcaneal osteotomy or the presence of neurological disease (e.g. autoimmune diseases), (ii) inflammatory arthropathy, gout, or rheumatoid arthritis; and (iii) coexisting midfoot or ankle deformities or previous surgery for correction of HV.

All procedures were performed in accordance with regulations and approved by the ethics committee of our hospital. Patients with symptoms including an HV angle ≥20°, an IMA >11°, and an incongruent first metatarsophalangeal joint were enrolled and underwent operations^8^. Metatarsalgia was recorded if the subject felt pain when walking with bare feet.

The enrolled subjects were examined using a Siemens spiral 64‐slice multidetector scanner from Germany (Siemens Medical, Freistaat Bayern). The technical parameters were set as follows: 120 kV, 80–110 mAs, a pitch of 0.9, and an acquisition thickness of 1 mm. CT scans from the calcaneus to the phalanx were reviewed in each subject, and HV deformity was found in those patients. CT scans were acquired by means of isotropic acquisition, stored in medicine format, and analyzed by using relative image processing software (Mimics). All other osseous structures were required to be normal and complete. The HV side was chosen as our objective.

### *Mimics Evaluation*

The function of region and threshold growth processing in Mimics software was used to segment the contours. In our research, Hounsfield units of 226 (minimum) and 1600 (maximum) were used as the threshold of bone tissue. The reconstructed three‐dimensional images were used to investigate the metatarsal bones and the HV deformities in all planes.

**Fig. 1:**
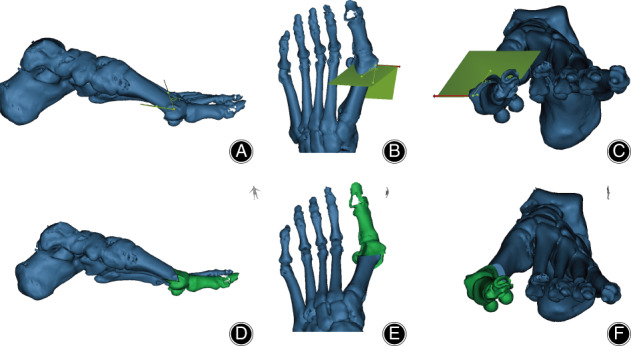
Three‐dimensional illustration of traditional Chevron osteotomy in Mimics. (A, D) The sagittal view of the Chevron osteotomy. (B, E) The transverse view of the Chevron osteotomy. (C, F) The coronal view of the chevron osteotomy.

**Fig. 2:**
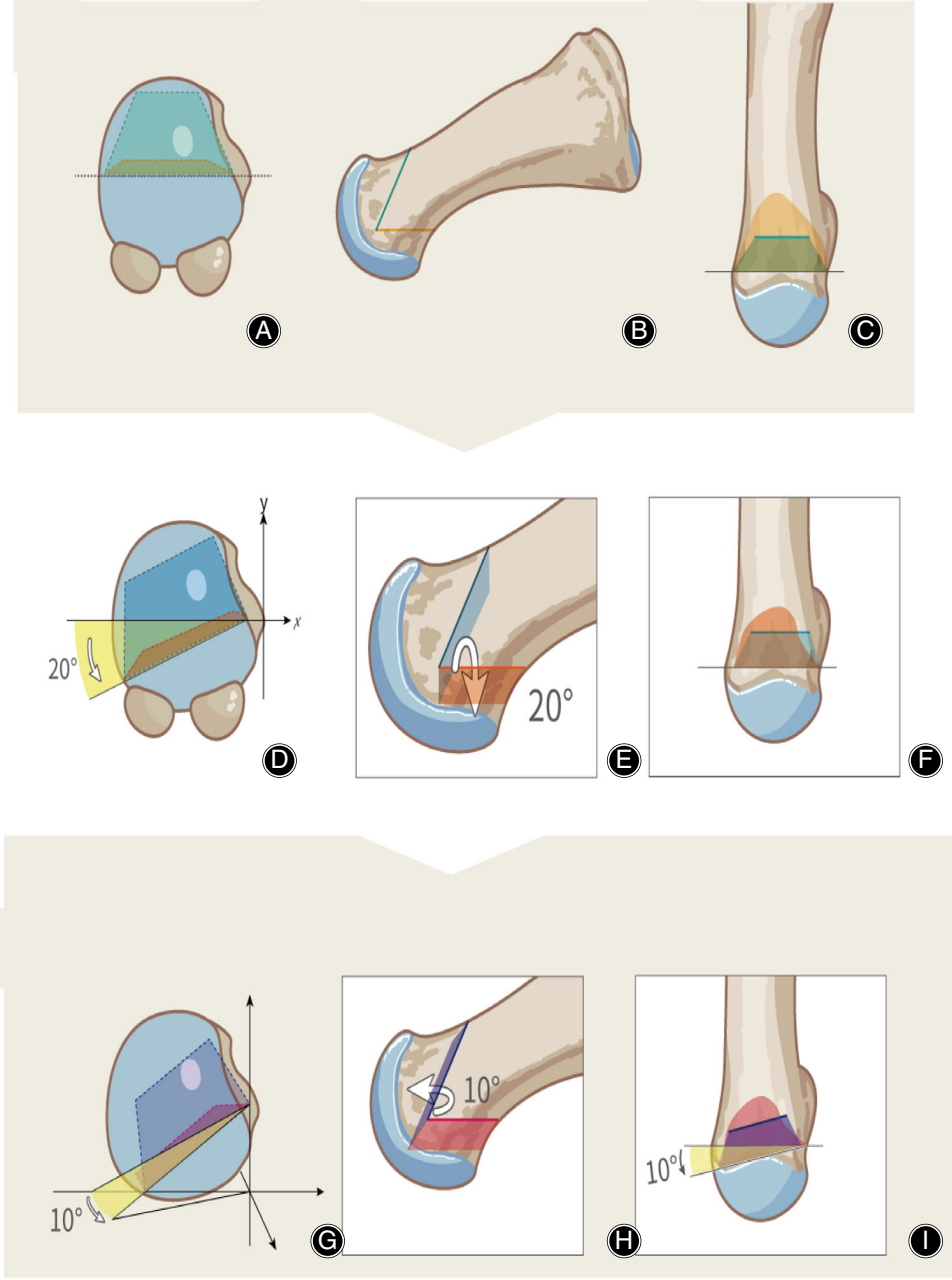
Schematic illustration of traditional chevron, planar, and triplanar osteotomy. (A, B, C) The coronal, sagittal, and tranverse view in chevron osteotomy. (D, E, F) The coronal, sagittal, and tranverse view in planar osteotomy. (G, H, I) The coronal, sagittal, and tranverse view in triplanar osteotomy.

**Fig. 3:**
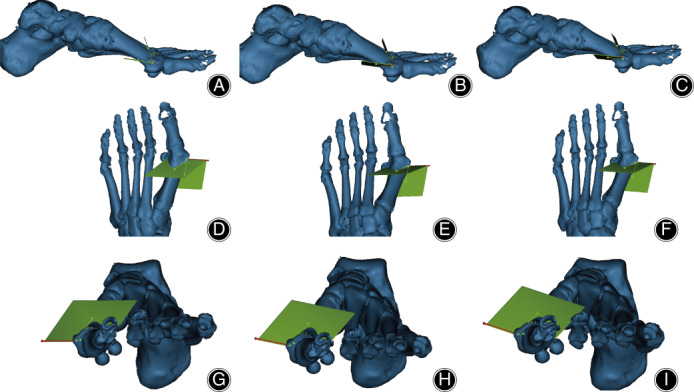
Three‐dimensional illustration of traditional Chevron, planar, and triplanar osteotomy in Mimics. (A, D, G) The sagittal, tranverse, and coronal view of the chevron osteotomy. (B, E, H) The sagittal, tranverse, and coronal view of the planar osteotomy. (C, F, I) The sagittal, tranverse, and coronal view of the triplanar osteotomy.

The standardized or modified cut planes were created and mimicked as follows. First, the apex for the chevron osteotomy procedure was located at the center of the first metatarsal and was defined as the line formed by the central point perpendicular to the fourth metatarsal bone (Figs 1 and 2A–C). Second, the cut planes of the POCO were defined as follows: chevron osteotomy along with 20° of plantarward obliquity (Fig. 2D–F). Third, the triplanar osteotomy incision was made using the POCO method, with the direction inclined by 10° distally (Fig. 2G–I). The relative linear and angular measurements were conducted using the Mimics workstation. The images of the planes of osteotomy for the three methods are presented in Figs 2 and 3.

**Fig. 4:**
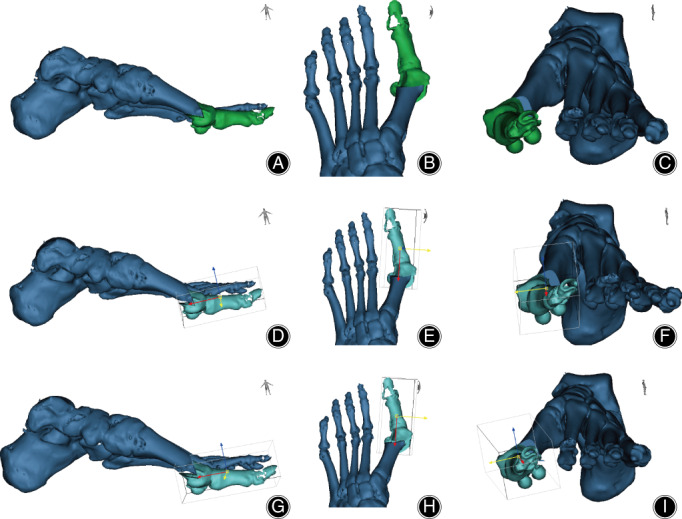
Three‐dimensional illustration of traditional Chevron, planar, and triplanar osteotomy in Mimics after osteotomy. (A, B, C) The sagittal, tranverse, and coronal view after chevron osteotomy. (D, E, F) The sagittal, tranverse, and coronal view after planar osteotomy. (G, H, I) The sagittal, tranverse, and coronal view after triplanar osteotomy.

Then, the cutting planes were simulated with the cutting button in Mimics. The osteotomy position and angle were determined, and the distal and proximal fractures were separated (Fig. 4). To mimic deformity correction, the distal fragment was laterally translated, and the first metatarsal head was unchanged in normal chevron osteotomy but lowered in plantar and triplanar osteotomy^6^. The distal fracture was placed laterally, and then the axial, sagittal, and coronal pictures were illustrated. The three methods were all performed and differences were observed.

### *Measurement of Hallux Valgus Deformity*

Relative outcome measures, such as the HV angle (HVA) and the intermetatarsal angle (IMA), were determined using standard weight‐bearing radiographs. The HVA was the angle created by the intersection of the proximal phalanx and the longitudinal axis of the first metatarsal. The intermetatarsal angle (IMA) of the first–second metatarsal was named as the angle created by the intersection of the longitudinal axis of the second and first metatarsals.

### *Measurement of Length of the First Metatarsal*

**Fig. 5:**
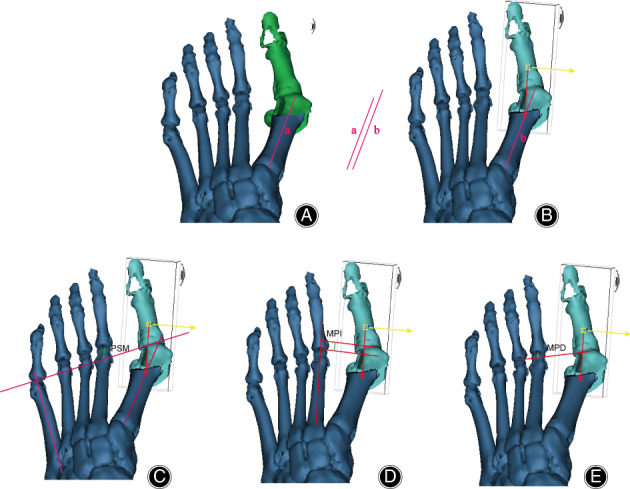
Illustration of the measurement method for relative degree in the first and second metatarsal with Mimics. (A, B) The traditional measure of the first metatarsal in chevron and Triplanar osteotomy. (C) The measurement method for projection of the second metatarsal (PSM). (D) The measurement method for metatarsal protrusion index (MPI). (E) The measurement method for MPD.

**Fig. 6:**
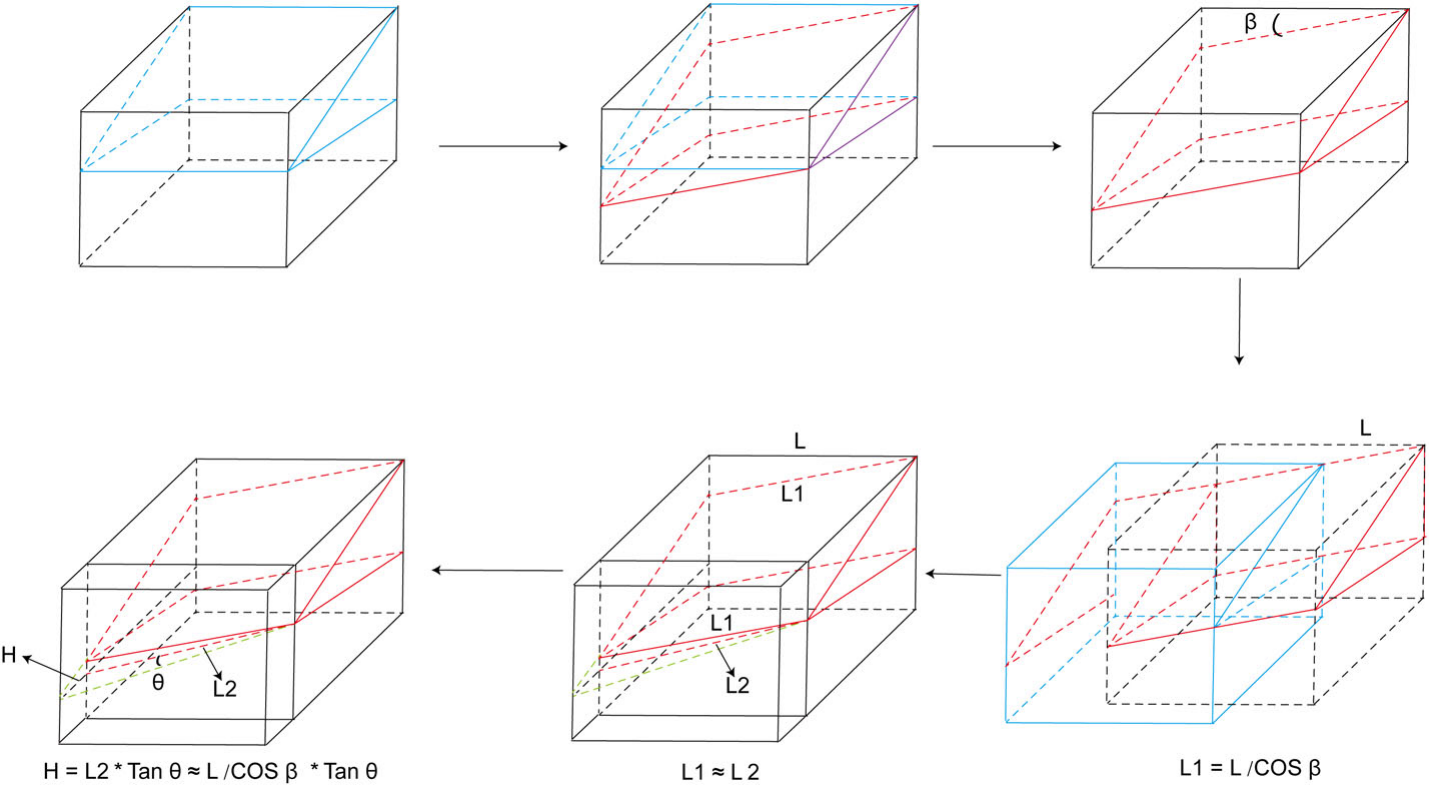
Calculated equation of increased length of the first metatarsal with tripanar osteotomy. The illustration started with traditional chevron osteotomy, followed by planar osteotomy, and, finally, triplanar osteotomy. The location of wedge‐shaped bone osteotomy on the inner side of the distal end of the metatarsal should be kept at the same point. L represents the width of the first metatarsal and L1 represents the length of the dorsal osteotomy line. L2 represents the length of the long line of the triangle. β represents the planar degree in sagittal view; it was 20 in our research. θ represents the distal incline degree in transverse plane; it was 10 in our research. The length of L2 was calculated with the following equation: H = L2 * Tan θ ≈ L/COS β * Tan θ.

The length of the first metatarsal was measured within the Mimics workstation (Fig. 5). The increased length of the first metatarsal was also calculated and illustrated with an equation (Fig. 6).

### *Measurement of* *Second‐Ray* *Pathologies*

The projection of the second metatarsal (PSM), the metatarsal protrusion index (MPI), and the metatarsal protrusion distance (MPD) were measured as reported by Young^9^. The PSM is the distance formed by two identified lines. One line was drawn through the tops of the first and fifth metatarsal heads and the other line was formed by the axis of the second metatarsal and the top of the second metatarsal head. The mean projection of the second metatarsals was 11 mm.

The MPI was identified as the distance between tangents on the ends of the first metatarsal heads drawn perpendicular to the longitudinal axis of the second metatarsal. A negative index (−) means that the first metatarsal was shorter than the second, a positive index (+) means the first metatarsal was longer than the second. Fleischer suggested when the MPI was less than −4 mm, plantar plate pathology may be impacted, causing deformity of the second ray^10^.

The MPD is the distance drawn from the apex of the second metatarsal to a line concatenating the top end of the third and first metatarsals. Klein *et al*. (2013) reported that long second metatarsals were commonly observed when the MPD was used^11^. The longer the MPD, the greater risk of HV deformity.

#### *Statistical Analysis*

Means and standard deviations (mean ± SD) were calculated. All structures of the HV were measured individually. In this study, all measurement results were analyzed as quantitative variables. The statistical analysis was conducted with SPSS 21.0 (Version 21, IBM, Armonk, NY, USA). The Pearson χ^2^‐test and the paired *t*‐test were used to compare the differences between relative data. Fisher's exact test was performed when the expected frequency was less than 5. A two‐sided probability value of *P* < 0.05 means there was a statistically significant difference. A highly significant difference occurred when *P* < 0.01.

## Results

### *Measurement Results for Hallux Valgus Deformity*

A total of 59 feet from 49 patients meeting our inclusion criteria were included. The average age at the time of surgery was 49.6 years (range, 30–63 years). In our research, 53 patients had a painful plantar callosity, and painless metatarsalgia was observed in others before surgery.

#### *Measurement Results After Traditional Chevron Osteotomy with Mimics*

The results showed that the HVA was significantly decreased after surgery in the traditional group (32.7° ± 4.6° *vs* 14.5° ± 1.7°, *t* = 26.854, *P* < 0.001). The IMA was also significantly decreased compared with the results before surgery in the traditional group (14.7° ± 2.0° *vs* 8.1°±1.1°, *t* = 20.787, *P* < 0.001) (Table 1).

Table 1 The measurement results after traditional Chevron osteotomy to HV

**TABLE 1:** The measurement results after traditional Chevron osteotomy to HV

	HV	Traditional	*P*
HVA, degree	32.7 ± 4.6	14.5 ± 1.7	<0.001
IMA, degree	14.7 ± 2.0	8.1 ± 1.1	<0.001
PSM, mm	12.20 ± 1.39	12.20 ± 1.39	‐
MPI, mm	‐1.57 ± 0.27	‐1.57 ± 0.27	‐
MPD, mm	3.63 ± 0.87	3.63 ± 0.87	‐
Length of the first metatarsal, mm	58.86 ± 2.96	58.86 ± 2.96	‐

**TABLE 2:** The measurement results after POCO osteotomies to HV

	HV	POCO	*P*
HVA, degree	32.7 ± 4.6	14.5 ± 1.7	<0.001
IMA, degree	14.7 ± 2.0	8.1 ± 1.1	<0.001
PSM, mm	12.20 ± 1.39	12.20 ± 1.39	‐
MPI, mm	‐1.57 ± 0.27	‐1.57 ± 0.27	‐
MPD, mm	3.63 ± 0.87	3.63 ± 0.87	‐
Length of the first metatarsal, mm	58.86 ± 2.96	58.86 ± 2.96	‐

**TABLE 3:** The measurement results after Triplanar osteotomies to HV

	HV	Triplanar	*P*
HVA, degree	32.7 ± 4.6	14.9 ± 2.1	<0.001
IMA, degree	14.7 ± 2.0	8.0 ± 1.1	<0.001
PSM, mm	12.20 ± 1.39	7.22 ± 1.04	<0.001
MPI, mm	‐1.57 ± 0.27	0.52 ± 0.24	<0.001
MPD, mm	3.63 ± 0.87	0.53 ± 0.19	<0.001
Length of the first metatarsal, mm	58.86 ± 2.96	69.58 ± 3.35	<0.001

**TABLE 4:** The comparison about measurement results in three different osteotomies

	Traditional	POCO	Triplanar	*P*
HVA, degree	14.5 ± 1.7	14.5 ± 1.7	14.9 ± 2.1	0.072
IMA, degree	8.1 ± 1.1	8.1 ± 1.1	8.0 ± 1.1	0.336
PSM, mm	12.20 ± 1.39	12.20 ± 1.39	7.22 ± 1.04	<0.001
MPI, mm	‐1.57 ± 0.27	‐1.57 ± 0.27	0.52 ± 0.24	<0.001
MPD, mm	3.63 ± 0.87	3.63 ± 0.87	0.53 ± 0.19	<0.001
Length of the first metatarsal, mm	58.86 ± 2.96	58.86 ± 2.96	69.58 ± 3.35	<0.001

#### *Measurement Results After Plantarward Oblique Chevron Osteotomy with Mimics*

The results showed that the HVA was significantly decreased after surgery in the POCO groups (32.7° ± 4.6° *vs* 14.5° ± 1.7°, *t* = 26.854, *P* < 0.001). The IMA was also significantly decreased compared with the results before surgery in traditional and POCO groups (14.7° ± 2.0° *vs* 8.1° ± 1.1°, *t* = 20.787, *P* < 0.001). Assessment based on Mimics was conducted, and there were no differences in correcting deformity of HV in axial planes (HVA and IMA) with traditional osteotomy and POCO, respectively, (Tables 1 and 2).

#### *Measurement Results After Triplanar Osteotomy with Mimics*

Furthermore, the results showed that the HVA was significantly decreased after surgery in triplanar groups (32.7° ± 4.6° *vs* 14.9° ± 2.1°, *t* = 25.583, *P* < 0.001). The IMA was also significantly decreased compared with the results before surgery in the triplanar group (14.7° ± 2.0° *vs* 8.0° ± 1.1°, *t* = 22.739, *P* < 0.001) (Table 3). Compared with traditional osteotomy and POCO, there were no differences in correcting deformities on axial planes for the HVA (14.5° ± 1.7° *vs* 14.9° ± 2.1°, *t* = 1.835, *P* = 0.072, Table 4) and IMA (8.1 ° ± 1.1° *vs* 8.0° ± 1.1°, *t* = −0.97, *P* = 0.336, Table 4), but the triplanar osteotomy was superior in changing the length of the first and second rays.

### *Measurement Results for the Length of the First Metatarsal with Mimics*

The length of the first metatarsal was unchanged after traditional chevron and POCO osteotomies (Tables 1, 2, 4). However, there was a significantly increased length of the first metatarsal in the triplanar osteotomy group (58.86 ± 2.96 *vs* 69.58 ± 3.35, *t* = −45.221, *P* < 0.001, Table 4) compared with traditional chevron and POCO osteotomies. The average increased length of the first metatarsal was 10.9 ± 1.3 mm without considering bone loss during the operations (Table 4). Comparing these three methods, the superiority of the triplanar method was obvious. Metatarsalgia can be avoided more efficiently depending on decreased height of the first metatarsal head and increased length of the first metatarsal in triplanar osteotomy. Furthermore, the distal inclined degree in triplanar osteotomy can be adjusted to meet the need for deformity corrections.

### *Measurement Results for* *Second‐Ray* *Pathologies*

#### *Measurement Results for Projection of the Second Metatarsal*

No changes were observed for PSM after traditional osteotomy and POCO for HV deformity. Compared with traditional osteotomy and POCO, a statistically significant decrease was observed in terms of the PSM after triplanar osteotomy (12.20 ± 1.39 *vs* 7.22 ± 1.04 mm, *t* = 23.265, *P* < 0.001, Tables 3 and 4).

#### *Measurement Results for* *Metatarsal Protrusion Index*

No changes were observed for MPI after traditional and POCO osteotomy for HV deformity. There was a statistically significant decrease following surgery in terms of the MPI after triplanar osteotomy (−1.57 ± 0.27 *vs* 0.52 ± 0.24 mm, *t* = −49.381, *P* < 0.001, Tables 3 and 4).

#### *Measurement Results for Metatarsal Protrusion Distance*

No changes were observed for MPD after traditional osteotomy and POCO for HV deformity. The MPD improved from 3.63 ± 0.87 mm to 0.53 ± 0.1 mm postoperatively; there was a statistically significant difference for the MPD (3.63 ± 0.87 *vs* 0.53 ± 0.19 mm, *t* = 26.622, *P* < 0.001, Tables 3 and 4).

## Discussion

This study is the first to propose and comprehensively describe a new method of triplanar osteotomy for the treatment of HV. Through the method triplanar osteotomy workstation, the benefit of this method was obvious, and it provided useful and essential information about triplanar osteotomy for orthopaedic surgeons. The results illustrated that triplanar osteotomy could significantly decrease the degree of the deformity, as characterized by the HVA and IMA. The length of the first metatarsal was also increased significantly. The sustained length of the metatarsal was essential to rehabilitation and decreased the risk of HV reoccurrence. The research enriched our understanding of the morphology and pathology of HV, defined a safe range of hallux operations, and improved the understanding of HV for young clinical orthopaedic surgeons.

Hallux valgus is not only a deformity with medial deviation of the first metatarsal and lateral deviation of the sesamoids but also widely considered a triplanar deformity, with involvement of the transverse, sagittal, and frontal planes^6^, ^12^. The literature describes many treatment strategies for HV with the same degree of severity. Although correctional arthrodesis of the first tarsometatarsal joint can address all three plane deformities, most of the published literature has tended to focus on clinical and radiographic evaluations in the coronal plane. The most commonly used operation method in our department and others in China is chevron uniplanar osteotomy, so there is a high rate of recurrence of HV. To solve these problems, a modified POCO was proposed in our previous research. It corrected the deformity in the sagittal plane and had good follow‐up results, but it was observed that the length of the first metatarsal was shortened significantly after the operation^6^. The main reason for the shortening was the operation and bone remodeling process after osteotomy. In the published research, the average shortening of the first metatarsal was 5–10 mm after the operation for HV^13^, ^14^, ^15^. It was confirmed that the presence of metatarsalgia postoperation was caused by the shortened metatarsal. Although plantarward osteotomy can prevent metatarsalgia, an unchanged length of the first metatarsal is still found with this operation. Unsatisfactory rehabilitation can be avoided if the correction includes all three components^5^.

With increased shortening of the first metatarsal, the plantar pressure of the first ray decreases, while that of the lateral rays continues to increase. A consensus that a shortened first metatarsal during HV operations results in postoperative transfer metatarsalgia has been reached. However, Geng *et al*. (2019) report that a maximum shortening length of 6 mm increases the loading ratio of the central rays by 54.8%, and the result is considered to be within the safe range of the central rays^7^. They determined that the distal metatarsal segment can be pushed down sufficiently to compensate for non‐normal plantar force distributions. However, severe shortening does occur rather frequently (the bone loss from sawing) even if the osteotomy direction is controlled well. The safe length of 6 mm cannot be guaranteed in operations. In other words, when the first metatarsal is operated on, its changed or unchanged length should be guaranteed to ensure that the increased load does not reach the critical value for transfer metatarsalgia. Therefore, the length should be increased to some extent to offset the unavoidable decrease during HV operations. To solve this problem, triplanar osteotomy was performed with 20° of plantarward obliquity and an incline of 10° distally, as illustrated in Fig. 5. Triplanar osteotomy was a good choice to preserve or increase the length of the first metatarsal. Our research found that after the operation, the length of the bone increased by an average of 10 mm (without considering the absorbing bones in the software) when the distal incline was 10°. After surgeons are proficient in this procedure, the angle can be increased to meet the demand, and therefore, an increased definite length of the first metatarsal can be obtained as planned.

The incidence of HV deformity is higher in China, probably due to genetic susceptibility, race, and ethnicity. Chevron osteotomy is widely used for correction of HV in the first metatarsal bone, which decreases the abnormal increased IMA of HV deformities. Therefore, the method proposed in our research has essential value in revolutionizing the use of chevron osteotomy in our department. The results demonstrated that the HVA and IMA were significantly improved after surgery. The reason for the decreased results was that Akin osteotomy was not mimicked in the software. After the Akin test was conducted, the deformity was significantly corrected. These data were consistent with our previous findings that chevron plantar osteotomy combined with Akin osteotomy is an effective treatment strategy for HV.

Another important treatment method used in the clinic is scarf osteotomy, which allows horizontal displacement, rotation, elevation, and lowering of the metatarsal head for moderate to severe deformities. Scarf osteotomy also prevents shortening of the first ray and can achieve early mobilization in patients. A previous study confirmed that scarf osteotomy had better corrective ability for HV deformity than distal metatarsal osteotomy^16^. Jeuken compared chevron and scarf osteotomies and reported that there were no significant differences in outcomes between these two procedures^17^. However, research has also revealed that scarf osteotomy should be used with caution in the correction of symptomatic adolescent HV due to the high recurrence rate. Therefore, scarf osteotomy has its own limitations in treating HV. The results regarding the outcome of second‐ray pathologies after triplanar osteotomy in this research demonstrated excellent rehabilitation without changing the length of the second metatarsal by Weil osteotomy. With the merits of a short learning curve and relatively minimal invasiveness, although clinical application of the operation has not yet been achieved, its superiority to other methods is obvious, and it will be popular and widespread in a few years.

The first limitation is that we could not mimic bone loss in the operation or the healing process with Mimics. The second was that CT in HV patients was not examined routinely, and the number of patients was limited. In our future research, the newly proposed operation will be conducted in the clinic, and more patients will be enrolled. Third, hallux pronation was one of the frequently found components, especially in larger deformities, but the cause and exact location of this condition are not fully understood. HV has been reported to be an anatomical variation characterized by bone rotation toward pronation compared with patients without it. Although a nature ability of correcting rotation deformity does exist with Chevron osteotomy, the rotation of the first metatarsal toward pronation was not well corrected and monitored in our research. In our future studies, a new method of osteotomy that allows the distal metatarsal segment to be supinated (externally rotated) will be proposed.

In summary, the results demonstrate that modified chevron or triplanar osteotomy can effectively correct HV deformities. It not only allows the ventral migration of the metatarsal head but also maintains or even increases the length of the first metatarsal and provides an opportunity for patients who may possibly need additional future deformity correction. Therefore, triplanar osteotomy is a safe and effective method to treat HV and reveals potential benefits of correction and prevention of transfer metatarsalgia in simulation experiments, with better efficacy and a short learning curve. In our future research, clinical application will take place to test the feasibility of this procedure.
